# Upper Mediastinal Lymphadenectomy Utilizing Prone-Position Thoracoscopy for Esophageal and Gastroesophageal Junction Cancers

**DOI:** 10.3390/jcm13226896

**Published:** 2024-11-16

**Authors:** Spyridon Davakis, Dimitrios Ziogas, Pavlos Papadakis, Stratigoula Sakellariou, Athanasia Mitsala, Christos Tsalikidis, Alexandros Charalabopoulos

**Affiliations:** 1 Upper Gastrointestinal and General Surgery Unit, First Department of Surgery, National and Kapodistrian Universtity of Athens, Laiko General Hospital, 11527 Athens, Greece; 2 First Department of Internal Medicine, National and Kapodistrian University of Athens, Laiko General Hospital, 11527 Athens, Greece; 3 First Department of Pathology, School of Medicine, National and Kapodistrian University of Athens, 11528 Athens, Greece; 4 Second Department of Surgery, Faculty of Medicine, Democritus University of Thrace, 68100 Alexandroupolis, Greece

**Keywords:** esophageal cancer, adenocarcinoma, minimally invasive esophagectomy, prone position thoracoscopy, lymph node dissection, superior mediastinal lymph nodes

## Abstract

**Background/Objectives:** Esophagectomy is the mainstay of treatment in esophageal cancer. Minimally invasive esophagectomy (MIE) remains a challenging procedure and has been associated with a high rate of complications and mortality. Routine lymphadenectomy includes two-field lymphadenectomy for distal-esophageal or gastroesophageal junction Siewert I–II tumors. Superior mediastinal lymphadenectomy (SML) refers to an extended two-field lymphadenectomy or total mediastinal lymphadenectomy during MIE for cancer. The exact benefits of SML have been the subject of prolonged debate, with no conclusive evidence indicating improved clinical and oncological results. Herein, we aim to present our surgical technique of thoracoscopic SML during MIE in the prone position, with short-term clinical and oncological outcomes. **Methods:** About 150 consecutive patients underwent totally MIE within 3 years period (2016–2019). SML included right-paratracheal nodes and nodes along the right-recurrent laryngeal nerve throughout its mediastinal route in cases of extended two-field lymphadenectomy, as well as left-paratracheal nodes and nodes along the left recurrent laryngeal nerve during total mediastinal lymphadenectomy. Eligible patients underwent SML during two-stage or three-stage MIE. **Results:** Twenty consecutive patients underwent SML during the study period. The 30- and 90-day mortality rates were 0. Pulmonary complications were observed in 16.5% of the patients. There was 1 right recurrent laryngeal nerve palsy noted. The median length of stay was 9 days. The median number of resected lymph nodes was 45, with the median SML nodes count being 8. The median follow-up was 24 months. **Conclusions:** SML during prone position thoracoscopy for esophageal cancer is safe and feasible, although technically demanding. Minimally invasive esophagectomy with SML may offer meaningful benefits in oncological outcomes without introducing additional significant morbidity. Further comparative studies are needed to better elucidate our results.

## 1. Introduction

Treatment and surgical management of esophageal/esophagogastric junction cancer remains a challenge, carrying high rates of peri-operative morbidity and mortality [1]. Over the past decades, minimally invasive esophagectomy (MIE) has become safe and feasible, reducing postoperative respiratory and cardiac complications and length of hospital stay, while offering favorable clinical and histopathological outcomes in the short- and long-term [2]. MIE techniques have advanced considerably from the initial hybrid approach, which combined thoracoscopy with laparotomy, to the current method, performed exclusively through laparoscopy and thoracoscopy [3,4]. Although MIE is technically demanding and has been correlated with a significant learning curve, it has evolved into a well-established surgical approach for the treatment of esophageal and esophagogastric junction tumors [5,6,7,8]. It is well known that the surgical anatomy of the upper mediastinum is thoroughly visualized and comprehended during MIE because of the significant magnification of the anatomical structures utilizing the high-resolution thoracoscopes [9]. Understanding the anatomy at this level ensures appropriate lymph node dissection and plays a key role in achieving long-term disease-free survival.

The International Society for Diseases of the Esophagus (ISDE) described different types of mediastinal lymphadenectomy in 1996 [10] that are still in use, with an updated definition in 2003: (I) standard: lymph node dissection up to the level of the carina (subcarinal nodes), (II) extended type: further nodal dissection—including right paratracheal lymph node clearance, (III) total mediastinal: involving both right- and left-paratracheal nodes, as well as both recurrent-laryngeal nerves lymphadenectomy and (IV) three-field lymph node dissection [11]. Furthermore, The European Society for the Diseases of the Esophagus (ESDE) has reached no consensus on the extent of mediastinal lymphadenectomy needed in the different types of esophageal and gastroesophageal cancers. Predominantly due to the location of the primary esophageal cancers, extended two-field lymphadenectomy, is not uncommon in squamous cell carcinomas, whereas few reports exist that justify this level of lymphadenectomy in the treatment of esophageal and gastroesophageal adenocarcinomas [12].

In this study, we present our experience in extended two-field and total mediastinal lymphadenectomy utilizing thoracoscopy in the prone position, in patients who underwent two-stage or three-stage MIE for esophageal and esophagogastric junction Siewert type I–II tumors.

## 2. Materials and Methods

### 2.1. Study Material and Design

This is a retrospective analysis of prospectively collected data over 5 years. About 160 consecutive two-stage or three-stage MIEs for cancer were performed from 2016 to 2021. Of these cases, 140 were two-stage while the remaining 20 were three-stage esophagectomies, performed under the same principles. This study took place at the Regional Esophago-Gastric Cancer Centre in Essex, UK and Upper Gastrointestinal and General Surgery Unit of the First Department of Surgery in Athens, Greece.

This study included only adult patients (>18 years) who were diagnosed with esophageal or gastroesophageal junction Siewert type I–II tumors. All of the enrolled patients had clinical disease to the upper mediastinal lymph nodes pre-operatively. All patients were treated with total MIE combining laparoscopic abdominal and thoracoscopic thoracic phase in a prone position; extension of lymphadenectomy included extended two-filed lymphadenectomy (abdominal D2—lower mediastinal—para-aortic—para-esophageal—subcarinal + right upper mediastinal lymph nodes) or total mediastinal lymphadenectomy (extended two-field + left upper mediastinal lymph nodes). Esophagectomies for benign disease as well as open procedures, emergency procedures, or procedures for malignant disease were excluded from the study. Additionally, all hybrid esophagectomies (laparoscopic/open thoracotomic) or cases in which conversion to open was needed were further excluded from this study.

All patients underwent preoperative staging, including Positron Emission Tomography–Computed Tomography (PET-CT), Endoscopic Ultrasound (EUS), and Computed Tomography (CT) of the chest and abdomen, as well as a Cardiopulmonary Exercise Test (CPET) to evaluate surgical fitness. Medical data were collected by reviewing patients’ records, followed by a personal examination and data registration during the postoperative follow-up period.

### 2.2. Statistical Analysis

Descriptive statistical analysis was performed for all measured parameters, with accumulated values presented as mean ± standard deviation, along with the corresponding range. Survival rates were assessed using the Kaplan–Meier survival estimator. All analyses were conducted using SPSS version 22.0 (Statistical Package for the Social Sciences Inc., Chicago, IL, USA).

### 2.3. Surgical Approach

#### 2.3.1. Two-Stage Totally MIE

**Figure 1:**
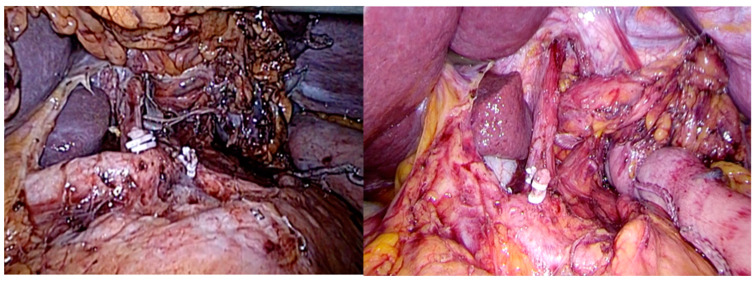
Abdominal D2 lymphadenectomy.

**Figure 2:**
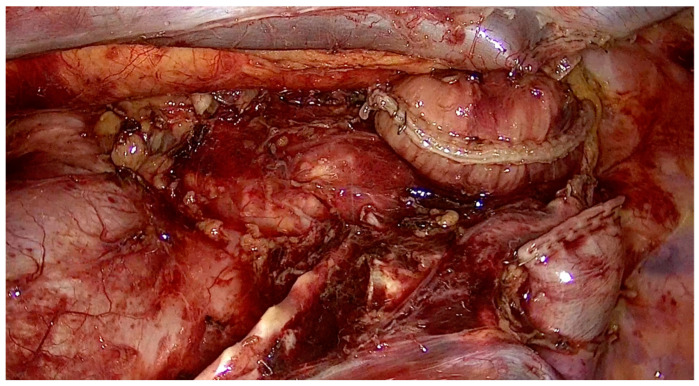
Standard thoracic lymphadenectomy.

This procedure stands for a laparoscopic abdominal phase followed by a thoracic phase utilizing a prone position thoracoscopy. The abdominal phase includes laparoscopic gastric mobilization and D2 lymphadenectomy (Figure 1) and construction of the gastric conduit. D2 abdominal lymphadenectomy includes lymph node dissection of the right gastric, left gastric, common hepatic, hepato-duodenal ligament, proximal splenic-artery, celiac trunk, right- and left-paracardial, infra-diaphragmatic, and diaphragmatic hiatus nodes. Following that, the patient is turned into a prone position, and the thoracic esophagus is dissected up to the level of the azygos arch. Standard thoracic lymph node dissection includes all the subcarinal nodes, left- and right-bronchial nodes, lower-posterior mediastinum nodes, para-aortic nodes, and para-esophageal nodes (Figure 2). An intrathoracic, hand-sewn esophagogastric anastomosis was performed in two layers thoracoscopically with all patients in the prone position.

#### 2.3.2. Three-Stage Totally MIE

This procedure refers to a three-stage esophagectomy performed with a thoracoscopic-thoracic phase in the prone position, followed by a laparoscopic-abdominal phase, as presented in the two-stage MIE, and cervical anastomosis during the final cervical phase of the operation. Standard two-field lymphadenectomy (thoracic lymphadenectomy up to the level of carina and D2 abdominal lymphadenectomy) is performed in all cases as in two-stage MIE, in the thoracic phase, and once esophageal mobilization and mediastinal lymph node dissection was performed, the patient was turned supine and a simultaneous abdominal and neck approach by two surgical teams was instigated. The thoracic phase was followed by a laparoscopic abdominal phase, as in the two-stage MIE, for stomach mobilization, conduit formation, and lymphadenectomy. During the cervical phase of the operation, the cervical esophagus was mobilized to enable communication with the right-chest, while identifying and preserving the left recurrent laryngeal nerve in all cases. After the division of the esophagus and specimen removal, gastric conduit was brought up to the cervix; a hand-sewn two-layer esophagogastric anastomosis was conducted in all cases.

#### 2.3.3. En Block Mid- and Lower-Esophageal Mobilization and Lymphadenectomy

Patients were placed in a prone position with the operating table slightly broken at the mid-chest level to increase the intercostal space as much as possible. During this phase of the procedure, the operating surgeon and the first assistant were both standing on the patient’s right side. Three 12-mm ports were used (the first trocar was placed posterior to the angle of the scapula at the 5th intercostal space for the camera, a second one at the posterior edge of the scapula at 3rd intercostal space—right hand operating port, and finally one port at 8th intercostal space, posterior to the scapular line—left hand operating port) under direct vision (optical trocars). Division of the mediastinal pleura is performed on both sides of the esophagus; it should be started between the vagal trunk and the right main bronchus, followed by exposure of the azygos vein. This allows en bloc lymphadenectomy to include the subcarinal area and exposure to the thoracic pre-aortic lymphovascular tissue, which lies posterior to the azygos. The azygos vein was then divided using Hem-o-lok clips, in order to gain access to the esophagus and peri-esophageal lymph nodes. Following that, the thoracic esophagus was mobilized caudally towards the diaphragmatic hiatus; subsequent division of the right-inferior pulmonary ligament was performed. The thoracic duct is then identified and divided using Hem-o-lok clips, 2–3 cm above the hiatus in the right para-aortic area.

#### 2.3.4. Upper Mediastinal Lymphadenectomy in Prone Position

The proximal thoracic esophagus is mobilized off the membranous part of the trachea starting from its right side. The thoracic esophagus, the trunk of the right vagus nerve, and the right subclavian artery were then dissected, and the right recurrent laryngeal nerve was identified and preserved. In order to apply traction to the proximal thoracic esophagus, a trans-esophageal full-thickness 0/0 Prolene suture on a straight needle was placed and pulled out through a separate 2–3 mm incision paravertebrally in patients who underwent three-stage MIE, while it was performed with slight traction to the dissected thoracic esophagus or by the means of an umbilical tape during two-stage MIE.

**Figure 3:**
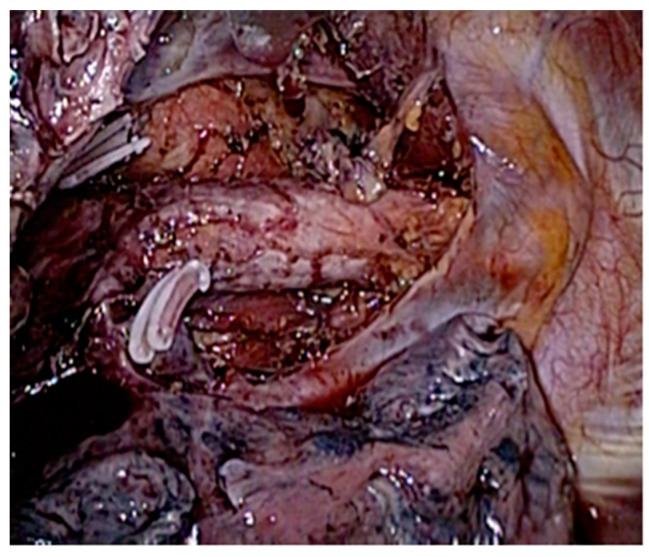
Superior mediastinal lymph node clearance (total).

**Figure 4:**
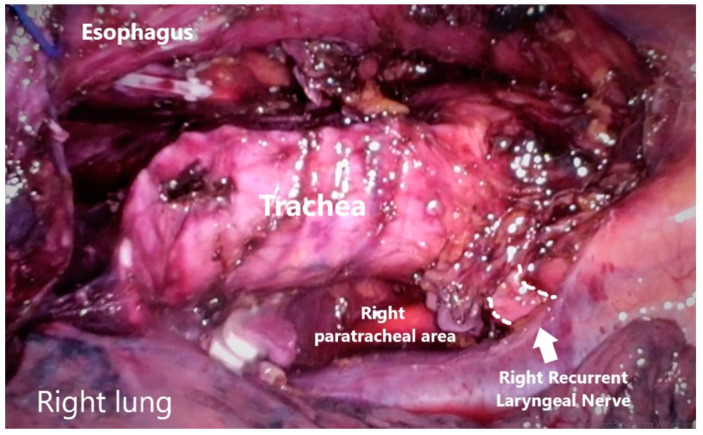
Superior mediastinal lymph node clearance (right).

**Figure 5:**
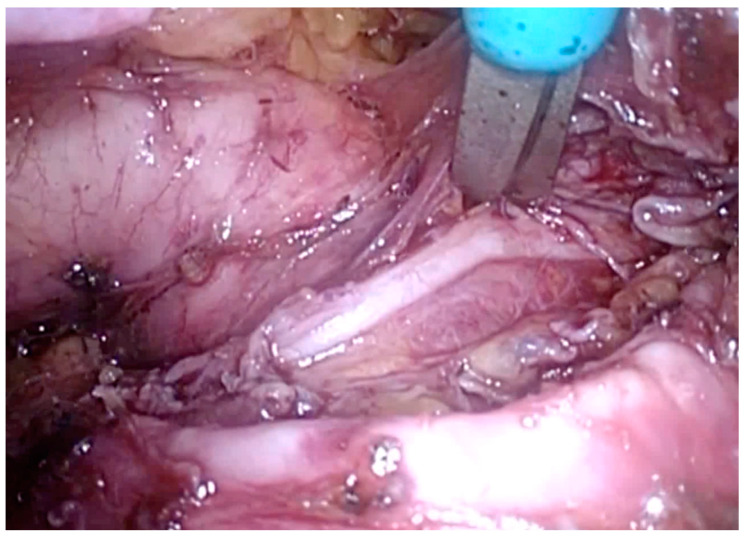
Left paratracheal nodes. LRN and aortic arch are visible left of the trachea and superiorly to the left main bronchus.

Right paratracheal nodes and nodes around the right recurrent laryngeal nerve were dissected. The proximal part of the thoracic esophagus is further mobilized cranially on its left-anterior side; an ample window is made after meticulous dissection of the space lying between the upper thoracic esophagus and trachea wall. With careful dissection, the left-recurrent laryngeal nerve was identified at the tracheoesophageal groove. Dissection of the infra-aortic and left tracheobronchial nodes is then performed. All of the lymphovascular tissues between the trachea and the left-recurrent laryngeal nerve were sharply dissected alongside the trachea and the left bronchus, in order to mark the upper border of dissection at the level of the thoracic inlet, completing thus the lymph node dissection along the left-recurrent laryngeal nerve (Figure 3, Figure 4 and Figure 5) (Video S1).

With patients placed in a prone position during the thoracic phase, both in two-stage or three-stage MIE, and the right lung deflated, there is no need for the use of an extra port for a pulmonary retractor; in all our cases, a single-lumen endotracheal tube (SLET) with bronchial blocker for the purpose of lung isolation and one lung ventilation instead of the double-lumen endotracheal tube (DLET). The devices used for dissection alongside the laryngeal nerves can be laparoscopic scissors without monopolar coagulation ablation or a laparoscopic energy device with a bipolar coagulation system to prevent thermal injury to the surrounding tissues. After completion of the esophageal mobilization and completion of the superior mediastinal lymphadenectomy on both sides, a 28-Fr chest tube was placed through the 12 mm incision at the 8th intercostal space, and the previously collapsed right lung was then expanded.

## 3. Results

During the study period, 14 patients underwent three-stage totally minimally invasive esophagectomy with total mediastinal lymphadenectomy and 6 patients underwent two-stage totally minimally invasive esophagectomy had extended two-field lymphadenectomy for esophageal or gastroesophageal junction Siewert type I–II cancer, with clinical disease to the superior mediastinal lymph nodes. All patients were treated between 1 January 2016 and 1 January 2021. The operative approach was identical between cases in each type of operation (2-stage MIE or 3-stage MIE).

All were male patients. Median age was 67 years (range: 58–75 years). Median operative time was 220 min for the two-stage MIE (range: 180–240 min) and 240 min for the three-stage MIE (range: 220–300 min). The median time for the thoracoscopic extended two-field lymphadenectomy was 30 min (range: 25–40 min) and 55 min for the total mediastinal lymphadenectomy (range: 50–65 min). Estimated blood loss was less than 250 mL in all cases. Four patients (*n* = 4, 25%) presented respiratory complications. There was one right-recurrent laryngeal nerve palsy (*n* = 1, 5%) noted in the postoperative period. The 30-day and 90-day mortality rates were 0. The median length of stay was 9 days (range: 8–11 days) and the median ICU stay was 1 day (range: 1–3 days).

The primary tumor location was the distal esophagus in 7 patients and the esophagogastric junction in 13 patients. Esophageal adenocarcinoma was the most common histopathologic type of cancer in 15 patients, while 5 patients were treated for squamous cell carcinoma of the esophagus. The median number of excised nodes was 45 (range: 22–116), while the median number of right paratracheal lymph nodes was 8 (range: 2–25) and the median number of left paratracheal lymph nodes was 6 (range: 2–10). Metastatic lymph nodes were identified in 3 patients (15%); 2 patients had positive right-paratracheal nodes while the remaining one had left paratracheal metastatic lymph nodes (Table 1).

## 4. Discussion

Esophageal cancer ranks as the sixth leading cause of cancer-related mortality worldwide, with subtle early symptoms often leading to advanced disease at the time of diagnosis [13,14]. Esophagectomy with extended lymphadenectomy for mid- or lower-esophageal cancer carries high morbidity and mortality rates. However, the utilization of minimally invasive techniques in recent years has improved surgical outcomes, particularly by reducing postoperative complications and hospital stays [14]. For the standard two-field lymph node dissection, peri-gastric lymphadenectomy and lower mediastinal lymphadenectomy up to the level of the subcarinal nodes are performed. Using minimally invasive approaches over recent years has improved surgical acuity, thus three-stage totally minimally invasive esophagectomy, including thoracoscopic thoracic phase followed by laparoscopic abdominal phase and cervical phase, is on the rise. Additionally, in selected patients with pre-operatively suspicious metastatic lymph nodes in clinical staging, utilizing PET CT and EUS [15,16,17], right upper mediastinal lymphadenectomy can be safely performed [15]. Despite these advances, complete upper mediastinal lymphadenectomy, including both left and right recurrent laryngeal nerves, is technically more demanding regardless of the use of open or thoracoscopic means.

The oncological effectiveness of minimally invasive upper mediastinal lymph node dissection for esophageal and gastroesophageal cancer is determined by its ability to achieve complete resection (R0 resection) and adequate lymphadenectomy. Multiple studies have demonstrated that minimally invasive approaches, particularly robotic-assisted techniques, can achieve superior lymph node yields, if not comparable, to open esophagectomy. Importantly, superior mediastinal lymph node dissection allows for en bloc resection of lymph nodes in the upper mediastinum, which is essential for achieving locoregional control, especially in squamous cell esophageal cancer, where upper mediastinal lymph node metastasis is more common than esophageal adenocarcinoma [18]. The extent of lymphadenectomy has been correlated with improved survival outcomes, particularly in SCC of the esophagus, where upper mediastinal lymph node metastasis is frequent. A study by Suda et al. indicated that extensive mediastinal lymphadenectomy during MIE, including upper mediastinal lymph node dissection, significantly improved overall survival and disease-free survival in patients with esophageal cancer. This suggests that upper mediastinal lymphadenectomy is a critical component of esophagectomy in these patients [19].

This study presents consecutive cases of a specific group of patients with esophageal and gastroesophageal junction Siewert type I–II tumors and clinical metastasis to the superior mediastinal lymph nodes. In this study, all surgeons felt that the thoracoscopic view in a prone position during the thoracic phase of MIE was significantly enriched with the glasses-based 3D-optical system using the 30°tip camera compared to the relevant 2D-optical system. As previously shown in other studies, so in ours, ultraprecise lymphadenectomy was made feasible, mainly due to vastly improved depth perception when utilizing the 3D camera system, thus potentially implying a significant improvement both in clinical and postoperative results of the patients undergoing MIE [20,21].

The thoracic phase of a three-stage minimally invasive esophagectomy in a prone position allows for better exposure and understanding of a clear and wide surgical view, omitting the need for special retractors or extra assistants. Nevertheless, surgeons can face significant technical difficulty in lymph node dissection along the left recurrent laryngeal nerve. To obtain a good exposure for lymph node dissection along the left-recurrent laryngeal nerve, slight and careful retraction only, or rotation, of the trachea toward the right side may be inadequate. Therefore, to further improve exposure during the procedure, we suggest breaking the table at the angle of the scapula. This can provide better exposure and access to the surgical field, particularly during the thoracic phase of the operation. Along with the prone position, we found that the utilization of a single-lumen endotracheal tube with a bronchial blocker allowed for better handling of the trachea compared to a double-lumen endotracheal tube. Despite known limitations of bronchial blockers, including poorer quality of lung collapse [22], more frequent tube malposition [23], and pressure exerted on the airway mucosa during prolonged surgery [24], our study found that the use of a bronchial blocker resulted in no major intraoperative complications related to ventilation, further supporting its utility in selected cases. Another important aspect to consider during the minimally invasive esophagectomy is the use of appropriate devices to minimize thermal spread to surrounding tissue. In our study, we successfully used a bipolar device with sealed jaws to reduce lateral thermal spread, which has been shown to be effective in previous studies [25].

Our findings suggest that thoracoscopic upper mediastinal lymphadenectomy during MIE in the prone position is technically feasible and safe, with promising early oncological outcomes. However, the small sample size and limited follow-up period preclude definitive conclusions about the long-term oncological benefits of this approach. Future prospective, multi-institutional studies with larger cohort sizes and longer follow-up periods are essential to better delineate the potential role of SML in improving the disease-free and overall survival rates in patients with esophageal and gastroesophageal junction cancers and clinical metastasis to the superior mediastinal lymph nodes. Moreover, further research should aim to refine the technical aspects of the procedure to minimize complications, particularly nerve injury, while maximizing the oncological efficacy of the lymphadenectomy.

## 5. Limitations

While the results of this study are promising, several limitations should be acknowledged. Foremost among these is the small cohort size. Additionally, the exclusion of hybrid and open procedures from this study further narrows the scope of applicability, as patients undergoing hybrid esophagectomy or requiring conversion to open surgery may result in different outcomes. The inclusion of both two- and three-stage MIE in the analysis, despite their inherent differences in complexity and extent of lymphadenectomy, may have introduced variability in the results that could not be fully accounted for in the analysis. Moreover, the retrospective nature of the study, despite the inclusion of consecutive patients treated by the same surgical team during the study period, inherently carries the risk of selection bias.

## 6. Conclusions

In summary, thoracoscopic upper mediastinal lymphadenectomy during minimally invasive esophagectomy (MIE) in the prone position demonstrates technical feasibility and safety, with promising early oncological outcomes for patients with mid-esophageal, distal-esophageal, and esophagogastric junction cancers. The use of a 3D-assisted optical system and the employment of precise surgical-energy devices improved our depth perception and minimized thermal spread, contributing to ultraprecise lymphadenectomy. Enhanced visualization, aided by the 3D system and patient positioning, allowed for better exposure to key anatomical structures, particularly during the superior mediastinal lymphadenectomy. However, challenges remain, particularly in dissection along the left recurrent laryngeal nerve; the use of laparoscopic scissors with no diathermy, good traction of the esophagus, and patency of the trachea with the use of single-lumen intubation and right bronchial blocker can significantly assist. Small sample size, exclusion of hybrid and open procedures, and inherent variability in two- and three-stage MIE procedures limit the generalizability of these findings. Additionally, the retrospective design and lack of long-term follow-up preclude definitive conclusions regarding the oncological benefits of this approach. Further prospective, multi-institutional studies with larger cohorts and extended follow-up periods are necessary to evaluate the long-term impact of this procedure on disease-free and overall survival. Future research should also focus on improving the technical aspects of the procedure to reduce complications and optimize the efficacy of upper mediastinal lymphadenectomy in esophageal cancer management.

## Figures and Tables

**Table 1:** Clinical characteristics and oncological outcomes.

Characteristics	Patients (*n* = 25)
Median age	67 years
Operative approach	
Two-stage totally MIE (Ivor Lewis)	6 (30%)
Three-stage totally MIE (McKeown)	14 (70%)
Extended two-field lymph node dissection (right paratracheal nodes)	8 (2–25)
Total mediastinal lymphadenectomy	6 (2–10)
Morbidity	5 (25%)
Laryngeal nerve palsy	1 (5%)
Median operative time—superior mediastinal lymph node dissection	Extended two-field: 30 min Total mediastinal: 55 min
Metastatic nodes/cases	3 (15%)

## Data Availability

All data are available on request.

## Supplementary Materials

The following supporting information can be downloaded at https://www.mdpi.com/article/10.3390/jcm13226896/s1, Video S1: Upper mediastinal lymphadenectomy utilizing prone position thoracoscopy.
